# Congenital extrahepatic portosystemic shunt type II occluded with cardiac closure device

**DOI:** 10.1016/j.radcr.2021.09.020

**Published:** 2021-10-07

**Authors:** Gisela Andrade, João Facas, Pedro Marques, Ana Nassauer Mónica, Paulo Donato

**Affiliations:** a Department of Radiology, Hospital Prof. Doutor Fernando Fonseca EPE, Amadora, Portugal; b Medical Imaging Department, Centro Hospitalar e Universitário de Coimbra, Coimbra, Portugal

**Keywords:** Portosystemic shunt, Gastrointestinal bleeding, Portal hypertension

## Abstract

Congenital extrahepatic portosystemic shunts are a rare cause of lower gastrointestinal bleeding in children. They result from the connection of a systemic vessel with the portomesenteric vasculature before the division of the main portal vein. Herein, we report a case of a congenital extrahepatic portosystemic shunts type II in a 4-year-old male diagnosed by Doppler ultrasonography during the investigation of abdominal pain and recurrent hematochezia, later confirmed by computed tomography. Conventional angiography with a balloon occlusion test revealed patent intrahepatic portal branches not depicted by previous imaging techniques. Successful shunt closure was achieved by endovascular approach with an Amplatzer Septal Occluder without complications.

## Introduction

A portosystemic shunt consists in an abnormal communication between the portal venous system and the systemic circulation, allowing venous flow to bypass the liver in a variable degree. Although most cases result from chronic portal hypertension (acquired shunts), they may also be of congenital origin [1].

Congenital extrahepatic portosystemic shunts (CEPS), also known as Abernethy malformations, are rare vascular anomalies that result from the connection of a systemic vessel with the portomesenteric vasculature before the division of the main portal vein (MPV) [1]. An anatomic classification described by Morgan and Superina in 1994 remains the most commonly used nowadays and divides them in two main types. A shunt with patent intrahepatic portal branches is defined as type II, whereas total shunts without any degree of liver perfusion are considered type I. Type I shunts may be further subclassified as Ib when the splenic and superior mesenteric veins join to form the portal vein or as Ia when they remain separate [2].

The clinical presentation of CEPS is highly variable, reflecting different anatomic configurations and coexistent pathologies. They may be diagnosed on neonatal screening tests due to high galactosemia or later in life after the development of complications, such as portal hypertension, hepatic encephalopathy, pulmonary hypertension and/or hepatopulmonary syndrome [3].

The initial presentation of CEPS may be secondary to associated congenital cardiac, abdominal and skeletal malformations, which often coexist [1,3]. There is also a known association between CEPS with benign and malignant hepatic lesions, which is thought to result from the compensatory increase of hepatic arterial flow [4].

## Case report

A 4-year-old male with a history of asthma was admitted to the emergency department with hematochezia and diffuse abdominal pain. His mother recalled two previous episodes of hematochezia in the last 5 months. Physical examination revealed facial telangiectasia and palmar erythema. His abdomen was soft and non-tender and no jaundice, fever, or peripheral edema were present.

**Fig. 1:**
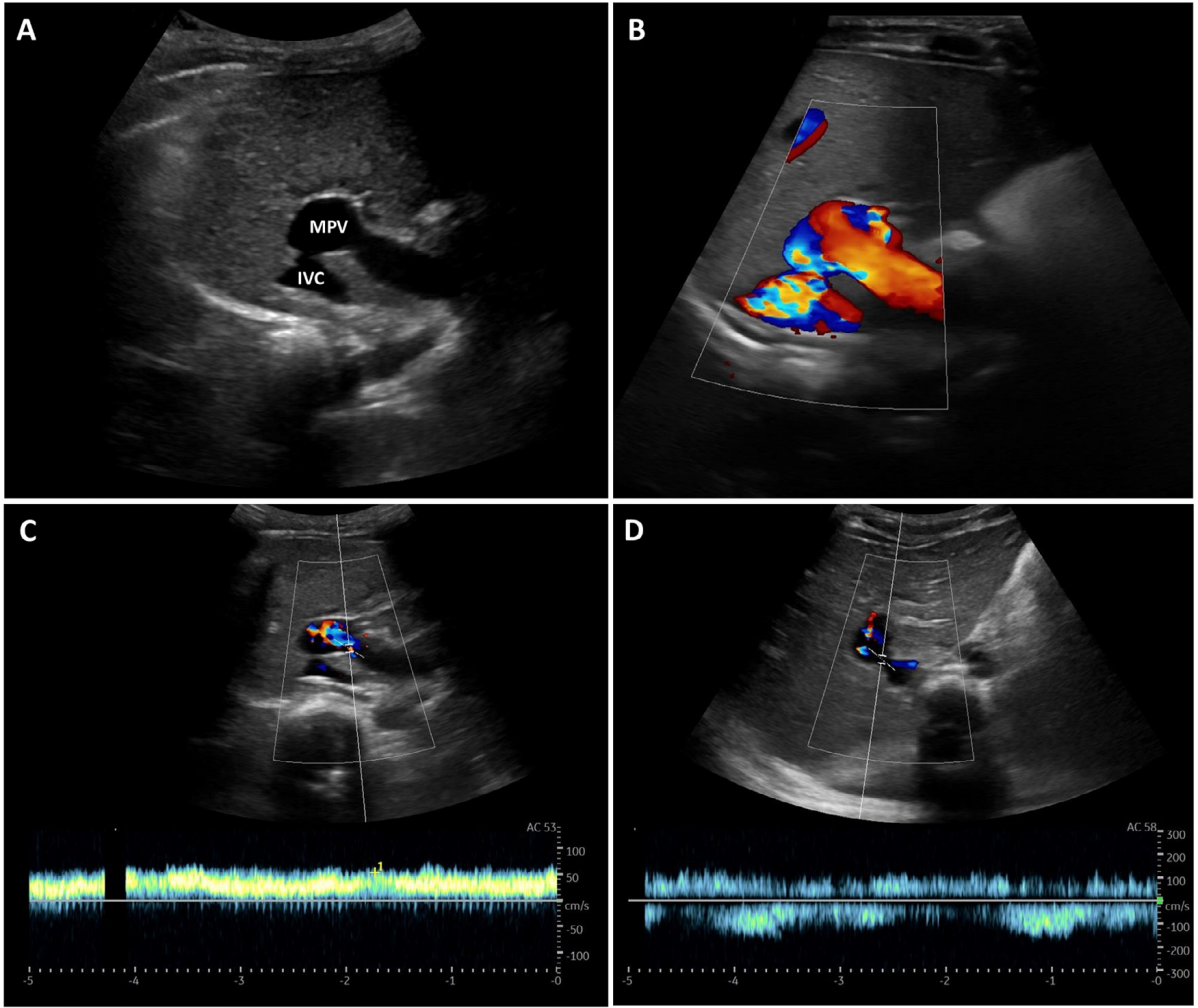
Ultrasonography with color and spectral Doppler. (A and B) Ultrasonography and color Doppler show an abnormal communication with flow between the MPV and the IVC. The MPV is enlarged, with a maximum diameter of 18 mm. (C) On spectral tracing the MPV has a normal monophasic and hepatopetal flow, but with high velocity (medium velocity of 54.9 cm/s). (D) Spectral analysis of the shunt depicts alternating hepatopetal and hepatofugal flow. IVC, inferior vena cava; MPV, main portal vein.

Laboratory evaluation showed normocytic normochromic anemia (hemoglobin of 9.4 g/dL,) but no other abnormalities in the remaining blood count and liver biochemical and function tests. An abdominal ultrasonography was performed to rule out intussusception. No distended or wall thickened bowel loops were noted. However, hepatic evaluation showed a direct communication between the MPV and the inferior vena cava (IVC) with flow on color and spectral Doppler (Fig. 1). No intrahepatic portal branches were seen on B-mode or Doppler. The MPV was enlarged, with a maximum diameter of 18 mm.

**Fig. 2:**
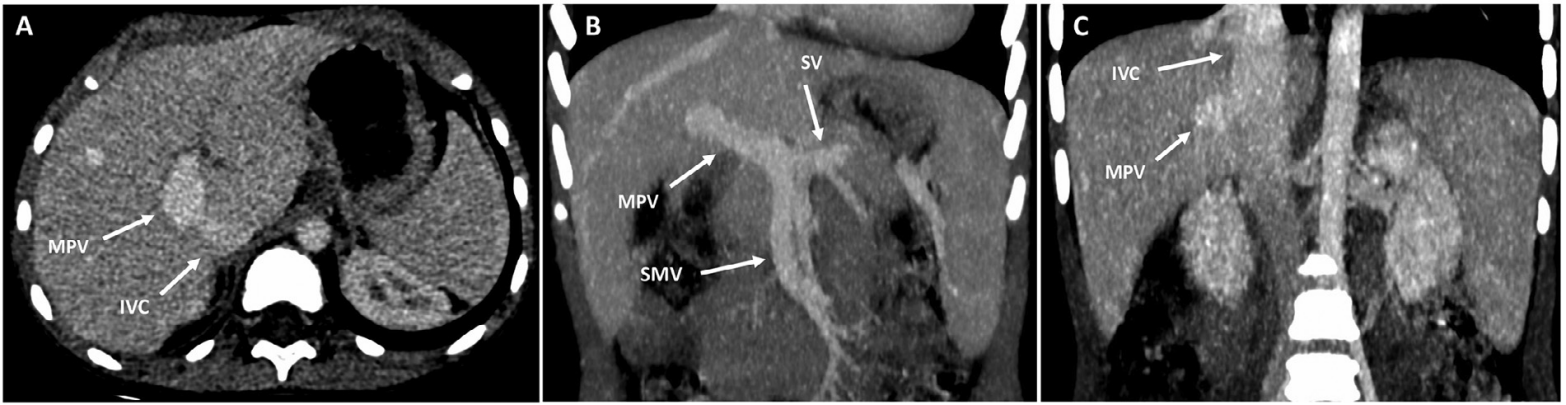
Abdominal CT in the portal venous phase. (A) CT in the axial plane shows a communication between the MPV and the IVC. (B and C) Maximum intensity projection (MIP) coronal reconstruction shows the MPV originating from the confluence of superior mesenteric vein (SMV) and splenic vein (SV) (B) and an anastomosis between the MPV and IVC (C). A homogeneous splenomegaly of 11 cm (long splenic axis) is also seen. CT, computed tomography; IVC, inferior vena cava; MPV, main portal vein.

Subsequent computed tomography (CT) angiography confirmed the presence of a portocaval shunt and showed a normal origin of the MPV, resulting from the confluence of the superior mesenteric vein and splenic vein (Fig. 2). CT also showed splenomegaly of 11 cm in length. The liver had a normal size, morphology and homogenous parenchyma, with no imaging findings of cirrhosis and no focal lesions. No other portosystemic collaterals were detected.

Liver magnetic resonance imaging (MRI) confirmed the absence of parenchymal lesions. Since no portal branches were detected by Doppler ultrasonography, CT and MRI, a type I CEPS was suspected.

Further investigation with colonoscopy found rectal varices and a bleeding varix was successfully sclerosed with polidocanol. Hemorrhoids and a nonbleeding anal fissure were also evident. Upper endoscopy was normal. Meckel diverticulum was ruled out on tc-99 m pertechnetate scintigraphy.

Serum alpha-fetoprotein and ammonia levels were normal. Transthoracic echocardiography and ventilation/perfusion scintigraphy did not reveal additional anomalies.

**Fig. 3:**
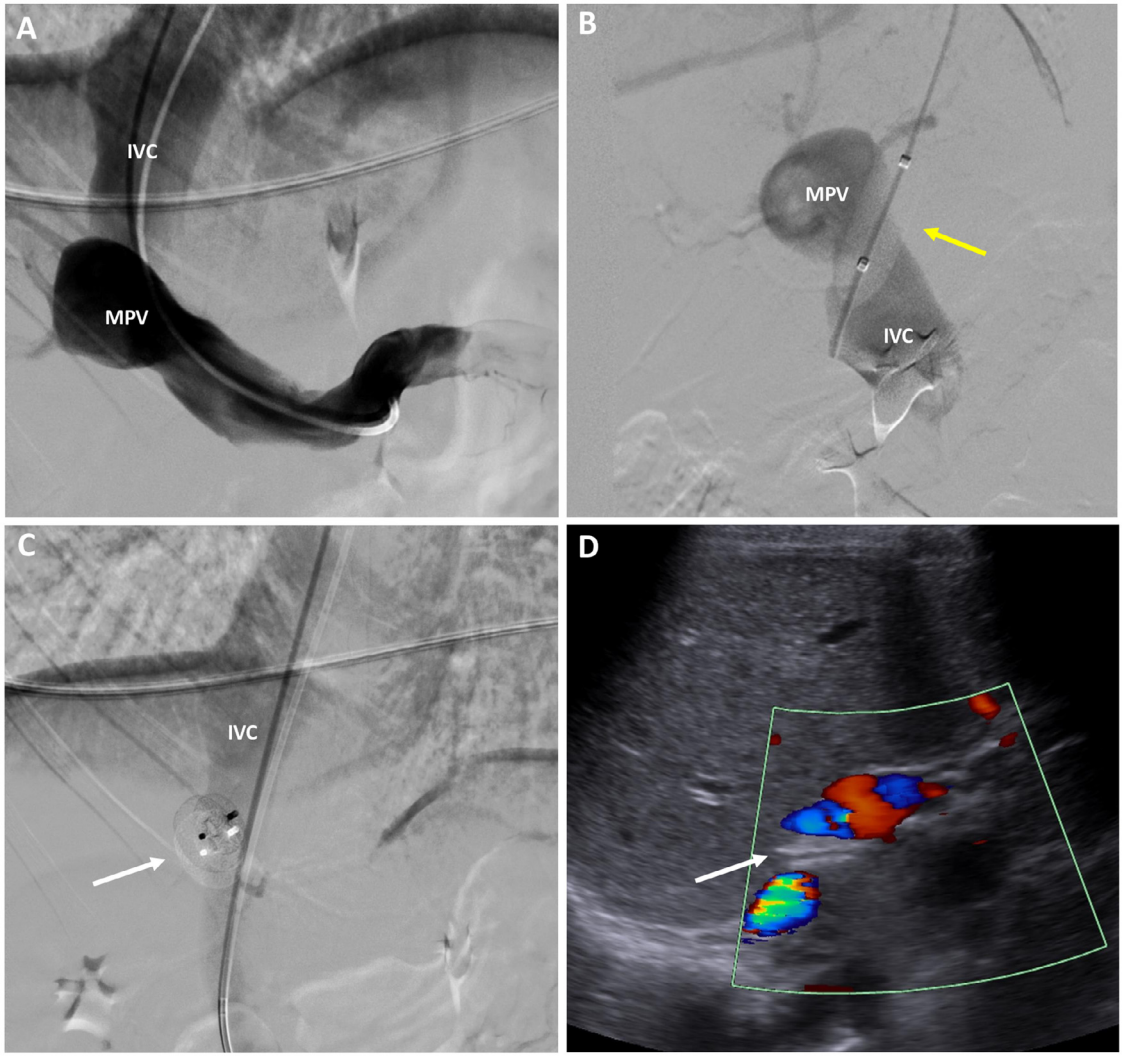
Transjugular portal venography and Doppler ultrasound. (A and B) Diagnostic venography with temporary shunt closure with a balloon (yellow arrow) shows opacification of the IVC, MPV and hypoplastic intrahepatic portal branches. (C) Therapeutic venography with deployment of a closure device (white arrow). (D) Color Doppler ultrasound performed 6 mo after device deployment demonstrates the device in the previous location of the portosystemic shunt (white arrow) and absence of flow between the MPV and IVC. IVC, inferior vena cava; MPV, main portal vein. “Color version available online.”

Portal venography with a percutaneous transjugular approach showed patent but hypoplastic intrahepatic portal branches, allowing the definitive diagnosis of CEPS type II (Fig. 3). Portal venous pressure was measured before and after temporary balloon occlusion of the shunt, with an initial pressure of 16 mmHg that went up to 21 mmHg after occlusion.

The shunt was closed 1 month later, in a single session, with an Amplatzer Septal Occluder (AGA Medical Corporation, Golden Valley, MN), without complications (disc diameters: 18 mm and 14 mm; waist diameter: 6 mm; waist width: 3 mm). A Doppler ultrasound of the liver performed 6 months later showed absence of flow between the MPV and IVC (Fig. 3). At 1-year follow-up the crisis of abdominal pain and anemia reverted but sporadic episodes of hematochezia still occur.

## Discussion

Herein we describe a type II CEPS with direct drainage of portal blood into the IVC. The shunt manifested itself as variceal lower gastrointestinal hemorrhage due to significant portal hypertension (16 mmHg).

Gastrointestinal hemorrhage is the presenting symptom in 8.1% of cases with CEPS and in 10.5% of CEPS with direct drainage to the IVC [5]. In CEPS associated with gastrointestinal bleeding, the most common systemic draining vessel is the iliac vein [5,6].

Cross-sectional imaging plays an important role in the work-up of these patients. Multidetector CT angiography is a valuable technique in the evaluation of the shunt anatomy due to its high spatial resolution [7], while MRI is the preferred imaging modality for detection and characterization of focal liver lesions [8].

Conventional angiography remains the best imaging technique for assessment of portomesenteric vasculature and allows the depiction of vessels before and after a shunt occlusion test. This temporary closure may reveal intrahepatic portal vessels not seen by other imaging techniques and it is thus essential for a correct classification of CEPS [9,10]. Measurement of portal pressure before and after the occlusion of the shunt should also be performed. The importance of conventional angiography is illustrated in this report, in which an incorrect type of CEPS was initially assumed by Doppler ultrasonography and contrast-enhanced CT.

The classification of CEPS has important therapeutic implications. Liver transplantation is the only curative treatment for type I shunts, while patients with preservation of some intrahepatic venous flow (type II) are candidates for surgical or endovascular shunt closure [1].

Shunt closure should be performed in type II shunts as early as possible after the first year of life, in order to prevent long-term complications [3]. A two-step approach, with reduction of the shunt size months before complete closure, is advocated when a high portal pressure is recorded during shunt occlusion (cut-off level described in the literature ranging from 25 to 32 mmHg) or when there is a pressure increase of at least 10 mmHg after shunt occlusion. When the occlusion pressure remains less than 25 mmHg and the pressure gradient less than 10 mmHg, the shunt can be safely closed in one session [9,11]. This was the case in this patient and the procedure took place without complications.

In conclusion, this report presents a rare cause of lower gastrointestinal bleeding in children. It also highlights how Doppler ultrasonography and cross-sectional imaging techniques are essential in the study of these patients but are not sensitive enough to rule out intrahepatic portal flow. Hence, management of CEPS should always include conventional angiography with shunt occlusion for an adequate classification and appropriate treatment.

## Patient consent statement

The authors declare that written informed consent was obtained from the patient's mother for publication of the case with accompanying images.
